# A Comparison of Molecular Typing Methods Applied to *Enterobacter cloacae* complex: *hsp60* Sequencing, Rep-PCR, and MLST

**DOI:** 10.20411/pai.v2i1.99

**Published:** 2017-02-01

**Authors:** Roberto A. Viau, Lee M. Kiedrowski, Barry N. Kreiswirth, Mark Adams, Federico Perez, Dror Marchaim, Dubert M. Guerrero, Keith S. Kaye, Latania K. Logan, Maria Virginia Villegas, Robert A. Bonomo

**Affiliations:** 1 Medical and Research Services Louis Stokes Cleveland Department of Veterans Affairs Medical Center, Cleveland, Ohio; 2 North Dakota State University, Fargo, North Dakota; 3 Public Health Research Institute Tuberculosis Center, Rutgers University, Newark, New Jersey; 4 J. Craig Venter Institute, La Jolla, California; 5 Department of Medicine, Infection Control and Prevention Unit of Infectious Diseases, Assaf Harofeh Medical Center; 6 Sanford Health, Fargo, Department of Infectious Disease, Fargo, North Dakota; 7 Wayne State University and Detroit Medical Center, Detroit, Michigan; 8 Rush University Medical Center, Chicago, Illinois; 9 Centro Internacional de Entrenamiento e Investigaciones Médicas (CIDEIM), Cali, Colombia; 10 Departments of Medicine, Microbiology and Cellular Biology, and Pharmacology, Case Western Reserve University School of Medicine, Cleveland, Ohio

**Keywords:** bacterial typing, molecular epidemiology, Enterobacter cloacae, infectious diseases outbreaks, rep-PCR, MLST, hsp60

## Abstract

Molecular typing using repetitive sequenced-based PCR (rep-PCR) and *hsp60* sequencing were applied to a collection of diverse *Enterobacter cloacae* complex isolates. To determine the most practical method for reference laboratories, we analyzed 71 *E. cloacae* complex isolates from sporadic and outbreak occurrences originating from 4 geographic areas. While rep-PCR was more discriminating, *hsp60* sequencing provided a broader and a more objective geographical tracking method similar to multilocus sequence typing (MLST). In addition, we suggest that MLST may have higher discriminative power compared to *hsp60* sequencing, although rep-PCR remains the most discriminative method for local outbreak investigations. In addition, rep-PCR can be an effective and inexpensive method for local outbreak investigation.

## INTRODUCTION

THe goal of molecular typing methods is to determine how closely bacterial strains are related. Typing methods can also help to determine the source of infection, trace transmission of healthcare pathogens, and to categorize strains with virulent behavior or decreased antimicrobial susceptibility [1]. An informative typing method must be able to differentiate between organisms with small genetic differences while having acceptable reproducibility, ease of performance, short turn-around time, and ease of interpretation [2]. In addition, the most useful typing method should be able to determine the extent of genetic differences encountered and help infer if evolution can be deduced from those differences.

For many years, pulsed field electrophoresis (PFGE) served as the “gold standard” for typing *Enterobacter spp.* [3]. Currently, PFGE, Enterobacterial repetitive intergenic consensus PCR (ERIC-PCR), repetitive element palindromic PCR (rep-PCR) [4], *hsp60* sequencing [5], and more recently, multilocus sequence typing (MLST) are being applied [6]. Other typing methods such as MLST [7] or whole genome single nucleotide polymorphism analysis [8] are also employed. However, PFGE is difficult to standardize between laboratories and challenging to interpret for laboratories with limited experience [9]. Methods based on whole genome sequencing provide reproducible results; however, whole genome sequencing data analysis is not yet fully standardized.

THe reproducibility of rep-PCR has been established for a wide range of pathogens, including *Klebsiella pneumoniae* [10], *Escherichia coli* [11] *Clostridium difficile* [12], and *Enterobacter cloacae* complex [4]. More recently, rep-PCR has been compared favorably to PFGE [4]. MLST, considered the “gold standard” for molecular typing of many species, has an unproven scheme for the discrimination of *E. cloacae* complex isolates [6]. MLST involves sequencing a DNA segment within 7 housekeeping genes, of which *hsp60* is not included. In comparison, *hsp60* sequencing possesses the advantage of being a single gene, overcoming the cost and labor required to perform MLST. In developing *hsp60* sequencing typing, Hoffman and Roggenkamp reported 12 clusters and 1 sequence crowd based on sequence divergences when describing the *E. cloacae* nomen-species [5]. In this report, we compare *hsp60* typing of *Enterobacter cloacae* complex to rep-PCR using a geographically diverse collection in order to gain insight regarding the most practical method to use in reference laboratories.

## MATERIALS AND METHODS

*Strains: Enterobacter cloacae* complex clinical isolates from a variety of sources (blood, urine, and sputum) were obtained from an outbreak in Fargo, ND (23); a pediatric hospital in Chicago, IL (21); an outbreak from Detroit, MI (8); and from Colombia in South America (20). Isolates were collected both during outbreaks and from sporadic cases. Species identification was confirmed with Vitek MS MALDI-TOF (RAB Laboratory).

hsp60 *sequencing:* Amplification of the *hsp60* gene was accomplished using previously described primers and conditions [5] using DNA isolated by heat lysis. DNA sequencing was performed at a commercial laboratory (MCLAB) and assembled with the Lasergene Software suite (DNASTAR) Seqman package. Pairwise distance was calculated via the Clustal W method and a cladogram was generated using Megalign. We inspected the cladogram for *hsp60* sequencing groups and then verified our findings using DNA alignment. Isolates were different if they had a single base difference at any position.

*rep-PCR*: DNA isolated with MO BIO Ultra Clean Microbial DNA isolation kit (Carlsbad, CA) was used for the *Enterobacter* spp. fingerprinting kit (bioMérieux, Marcy-L'Étoile, France) per the manufacturer's procedure. PCR products were separated by electrophoresis using microfluidic lab-on-a-chip (Agilent Bionalyzer 2100, Santa Clara, CA, USA). Results were analyzed using the Diversilab (bioMérieux) on-line software employing the Kullback-Leibler method, which places more emphasis on the presence or absence of bands than on their intensity[13].

*MLST*: Twenty-four of 71 representative isolates were selected to determine MLST type. DNA was isolated by heat lysis. Seven housekeeping genes were amplified according to the method by Miyoshi-Akiyama *et al* [6] using their described primer sets. Sequences were assembled using DNABaser (Heracel BioSoft, Romania). A neighbor-joining tree was generated from the allelic profiles using START2 [14].

*Data Analysis:* THe rep-PCR system was divided into 2 sub-groups. In the first sub-group we considered that all isolates with a similarity > 95% (according to the Kullback-Leibler method) belonged to the same strain. For the second group, we visually inspected the band pattern from isolates with >95% similarity and classified an isolate as different if we could identify at least 1 band difference.

Results from the 3 typing systems were compared using the Simpson's diversity index (SDI) to assess each method's ability to find differences between the isolates [15]. THe Adjusted Wallace coefficient was used to determine if the methods agreed with each other [16].

## RESULTS

All 71 isolates were analyzed by *hsp60* sequencing and rep-PCR. THe *hsp60* sequences were trimmed to include 316 base pairs. THus, *hsp60* sequencing could differentiate 25 unique isolates, and rep-PCR produced 36 unique partitions, while rep-PCR with manual review produced 47. SDI was 0.851 for *hsp60* sequencing, 0.933 for fully automated rep-PCR, and 0.975 for rep-PCR with manual re-interpretation (Table 1). Differences between all methods were statistically signifi-cant with *P* < 0.05 with one-tailed hypothesis.

**Table 1.** Simpson's Diversity Index for *hsp60* sequencing and rep-PCR with 72 isolates of *Enterobacter cloacae* complex

Method	Unique parti-tions	SDI	95% Confidence Interval
***hsp60***	25	0.851	(0.788-0.915)
**rep-PCR with manual re-classification**	47	0.975	(0.959-0.990)
**rep-PCR**	36	0.933	(0.897-0.969)

There is no statistical difference in the ability to discriminate between isolates when using *hsp60* or rep-PCR; or when using rep-PCR or rep-PCR with manual re-classification. However, rep-PCR with manual re-classification produced a significantly higher number of partitions than *hsp60* sequencing.

THe Adjusted Wallace coefficient with a value of 1 between rep-PCR and rep-PCR with manual re-classification showed that all groupings by rep-PCR with the Kullback-Leibler method were conserved after the manual inspection. THis value was 0.36 in the opposite direction, showing that only a fraction of classifications was left intact after the visual inspection. On the other hand, rep-PCR analysis by visual inspection or by the Kullback-Leibler method agreed with *hsp60* classifications with a coefficient of 0.459 and 0.416 respectively (*P* = 0.645). THe sequencing of *hsp60* only preserved rep-PCR partitions with a coefficient of 0.171 for rep-PCR with the Kullback-Leibler method and 0.068 after visual inspection using ≥ 1 band difference (*P* = 0.031) (Table 2).

**Table 2.** Adjusted Wallace Coefficient and 95% Confidence Intervals (CI) within *hsp60* sequencing and rep-PCR

	rep-PCR (95% CI)	Manual re-classification (95%CI)	*hsp60* (95% CI)
**rep-PCR**		0.361 (0.245-0.477)	0.416 (0.188-0.644)
**Manual re-classification**	1 (1)		0.459 (0.258-0.660)
***hsp60***	0.171 (0.051-0.292)	0.068 (0.002-0.135)	

Partition agreement between the tested typing methodologies shows the adjusted probability of a pair of isolates to be classified in the same group by rep-PCR if classified in the same group by rep-PCR with manual classification (0.36) or *hsp60* sequencing (0.42) (Not significant). Since rep-PCR with manual re-classification further divides rep-PCR partitions, if a pair of isolates is classified within the same group, it follows that they would be in the same group with rep-PCR alone (1). Meanwhile, the probability for two isolates in the same group by *hsp60* sequencing to remain together in the same partition when typed with rep-PCR with manual reclassification is of 0.46. On the other hand, the probability of a pair of isolates to remain in the same group when typed by *hsp60* sequencing is only 0.17 or 0.07 if they were in the same group when typed with rep-PCR or rep-PCR with manual reclassification, respectively.

## DISCUSSION

**Figure 1.**
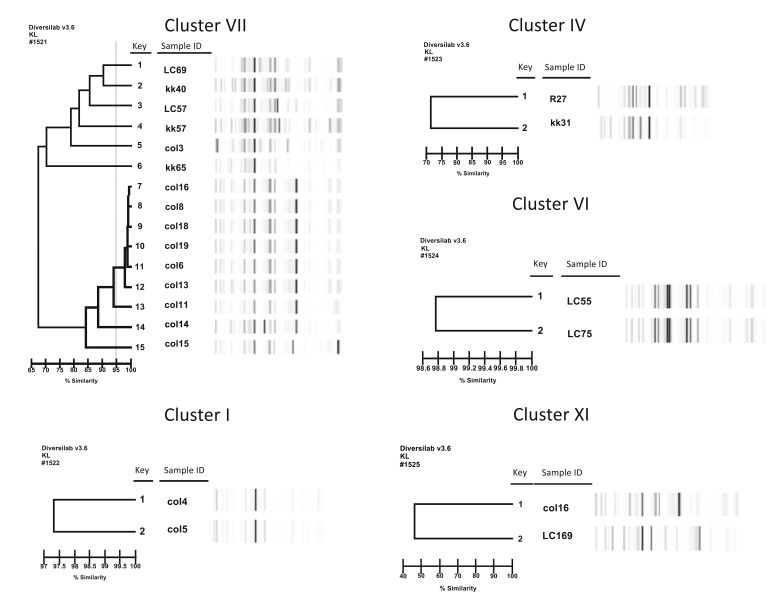
rep-PCR of selected samples by *hsp60* cluster

**Figure 2.**
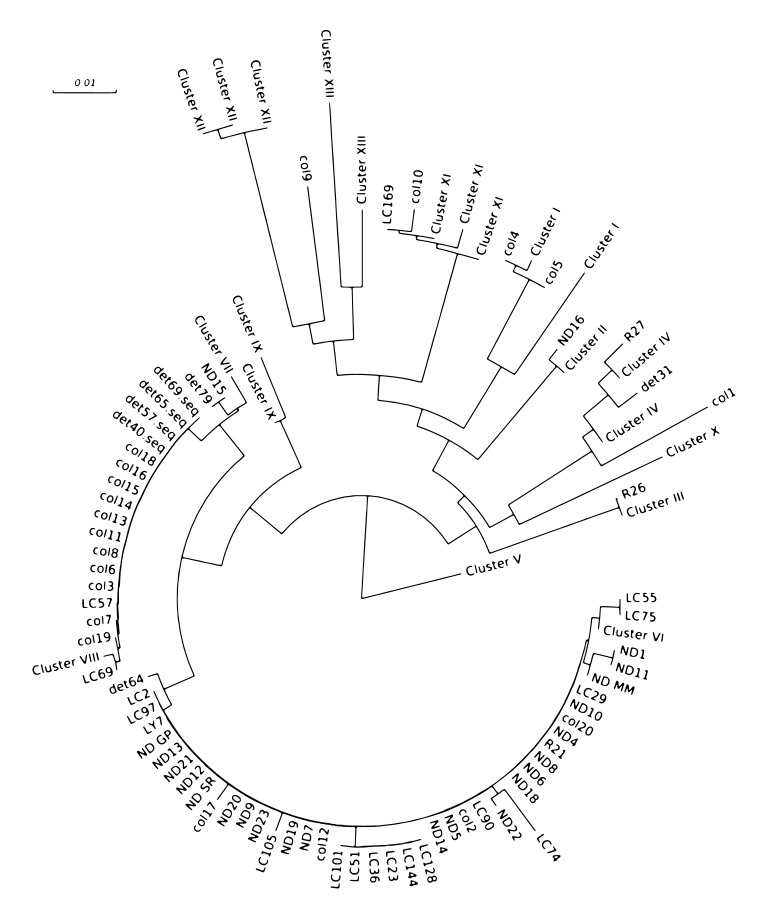
Phylogenetic tree by *hsp60* sequencing with classification by clusters

Our results further establish that rep-PCR can discriminate *E. cloacae* complex strains from diverse geographic origins. When compared to *hsp60* sequencing, we observed that rep-PCR shows a higher discriminatory ability. THe lower discriminatory ability of *hsp60* sequencing as revealed by the SDI (0.98 vs 0.85) is expected given that this is a highly-conserved gene. In contrast, rep-PCR amplifies repetitive areas distributed around the bacterial genome that can change because of chromosomal rearrangements. Despite this, compared to rep-PCR, *hsp60* sequencing was able to discriminate a comparable number of isolates. While most of the isolates acquired from patients belonged to clusters I, IV, VI, VIII, and XI of the Hoffman and Roggenkamp genetic clusters [5] we show that *hsp60* sequencing is able to provide greater resolution in differentiating isolates affecting humans than what can be provided only by *hsp60* cluster designation (Figures 1 and 2).

**Figure 3.**
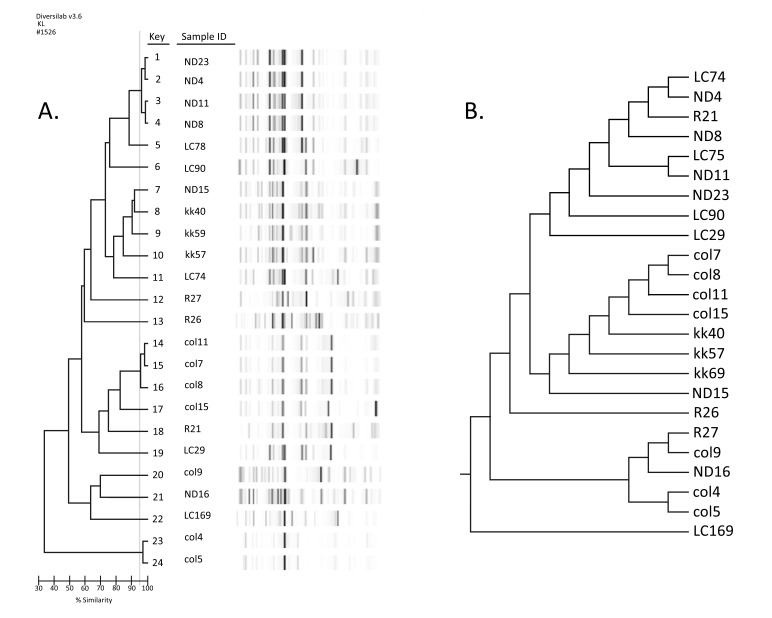
Isolates on which MLST was performed. A. Phylogenetic tree by rep-PCR; B. phylogenetic tree by *hsp60* sequencing

**Figure 4.**
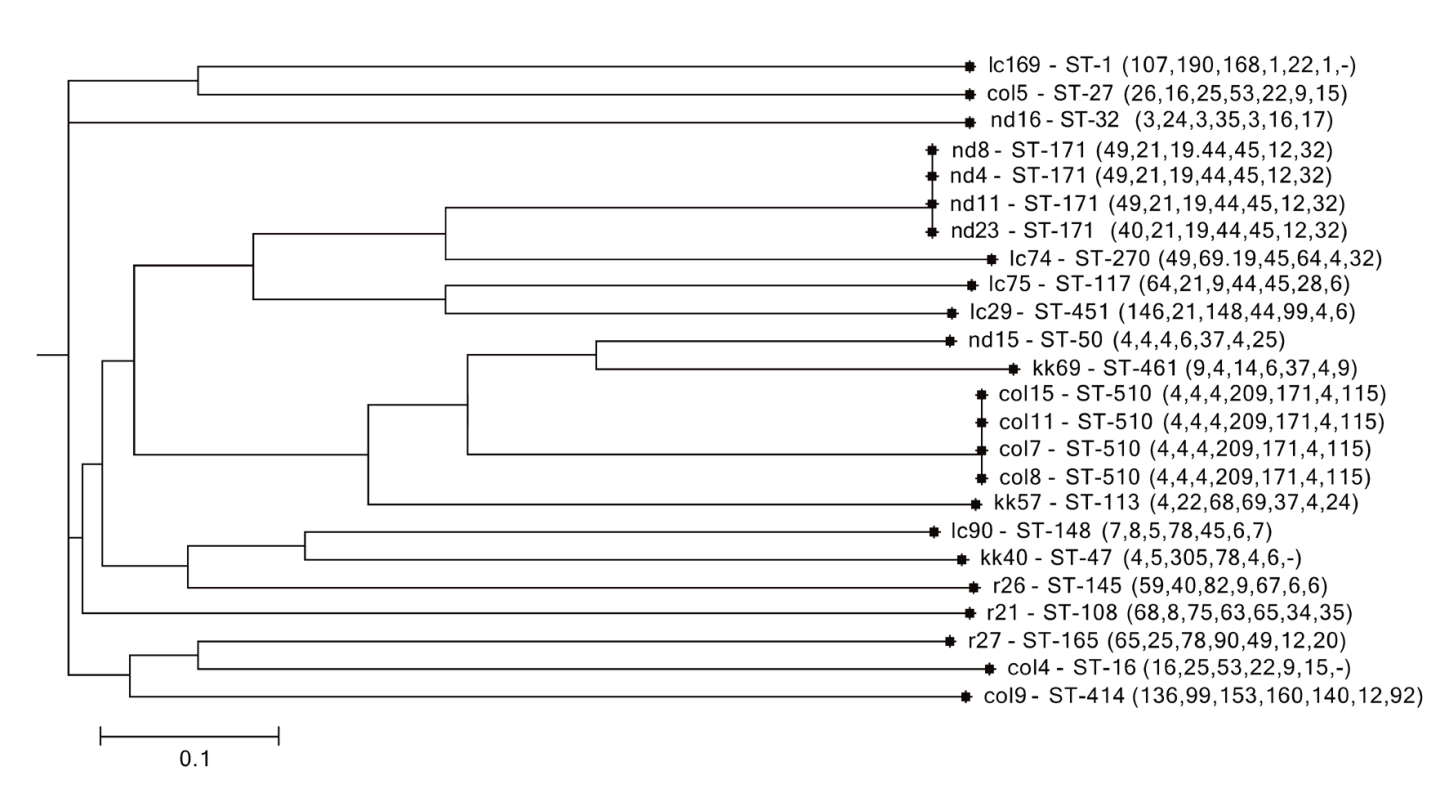
Phylogenetic tree by MLST

Analysis including MLST was available for 24 representative isolates (Figures 3 and 4, Tables 3 and 4). SDI was 0.924 for *hsp60*, 0.978 for rep-PCR, and 0.953 for MLST. After visual re-inspection, the rep-PCR index improved to 0.993. None of the differences between the index for MLST and the other methods reached statistical significance. Agreement within the different methods as measured by the Wallace coefficient show that all partitions detected by MLST were also detected by rep-PCR (Wallace coefficient of 1). However, not all partitions by rep-PCR were detected by MLST (Wallace coefficient of 0.45 and 0.148 for rep-PCR and rep-PCR with visual re-inspection, respectively). Sequencing of *hsp60* had a Wallace coefficient of 0.5 and 0.3 respectively, showing 50% of partitions by *hsp60* sequencing are also detected by MLST, but only 30% of those detected by MLST translate to different *hsp60* types. THese data further support the idea of rep-PCR being more capable of fine discrimination that the other typing methods assessed. In contrast MLST appears to be superior to *hsp60* sequencing.

**Table 3.** Simpson's Diversity Index (SDI) for rep-PCR, hsp60 sequencing, and MLST with 24 isolates of E. cloacae complex

Method	Unique partitions	SDI	95% Confidence Interval
***hsp60***	15	0.924	(0.859-0.989)
**rep-PCR with manual re-classification**	22	0.993	(0.980-1.000)
**rep-PCR**	19	0.978	(0.954-1.000)
**MLST**	17	0.953	(0.906-1.0000)

There is no difference in the number of groups achievable by any of the four typing modalities tested. THis is likely due to low number of isolates on which all four methods were performed.

**Table 4.** Adjusted Wallace Coefficient and 95% Confidence Intervals (CI) within *hsp60* sequencing, rep-PCR, and MLST

	MLST	rep-PCR (95% CI)	Manual re-classification (95% CI)	hsp60 (95% CI)
**MLST**		0.450(0.134-0.4765)	0.148 (0-0.450)	0.5 (0.126-0.875)
**rep-PCR**	1 (1)		0.328 (0-0.724)	0.459 (0.074-0.844)
**Manual re-classification**	1 (1)	1 (1)		0.459 (0-1)
**hsp60**	0.3 (0-0.609)	0.124 (0-0.345)	0.041 (0-0.243)	

A pair of isolates classified in the same group by rep-PCR, rep PCPR with manual re-classification, or *hsp60* sequencing has a probability of 0.45, 0.15, or 0.5 to remain in the same group when typed with MLST. Notice wide confidence intervals.

We conclude that the performance characteristics of rep-PCR make it suitable for epidemiological analyses such as outbreak investigations or determination of horizontal transmission in hospitals. We are also able to show that MLST provides finer classification than *hsp60* sequencing while not being as discriminative as rep-PCR. Furthermore, the relatively low agreement between *hsp60* sequencing and the other methods makes it less desirable for most applications, especially if used alone. Our findings indicate that rep-PCR may be a useful complement to other typing methods. THese results allow clinicians and epidemiologist to evaluate outbreaks of *Enterobacter cloacae* complex, which is of importance as drug resistance is becoming increasingly problematic.
